# Genome-wide association analysis explores the genetic loci of amino acid content in duck’s breast muscle

**DOI:** 10.1186/s12864-024-10287-1

**Published:** 2024-05-16

**Authors:** Rui Wang, Yinjuan Lu, Jingjing Qi, Yang Xi, Zhenyang Shen, Grace Twumasi, Lili Bai, Jiwei Hu, Jiwen Wang, Liang Li, Hehe Liu

**Affiliations:** 1 Farm Animal Genetic Resources Exploration and Innovation Key Laboratory of Sichuan Province, Sichuan Agricultural University, Chengdu, Sichuan P.R. China; 2 Key Laboratory of Livestock and Poultry Multi-omics, Ministry of Agriculture and Rural Affairs, Wenjiang District, 611130 Chengdu, Sichuan P.R. China; 3 National Key Laboratory for Swine and Poultry Breeding, Chengdu, P.R. China

**Keywords:** Duck, Breast muscle, Amino acid, GWAS

## Abstract

**Background:**

Amino acids are the basic components of protein and an important index to evaluate meat quality. With the rapid development of genomics, candidate regions and genes affecting amino acid content in livestock and poultry have been gradually revealed. Hence, genome-wide association study (GWAS) can be used to screen candidate loci associated with amino acid content in duck meat.

**Result:**

In the current study, the content of 16 amino acids was detected in 358 duck breast muscles. The proportion of Glu to the total amino acid content was relatively high, and the proportion was 0.14. However, the proportion of Met content was relatively low, at just 0.03. By comparative analysis, significant differences were found between males and females in 3 amino acids, including Ser, Met, and Phe. In addition, 12 SNPs were significantly correlated with Pro content by GWAS analysis, and these SNPs were annotated by 7 protein-coding genes; 8 significant SNPs were associated with Tyr content, and these SNPs were annotated by 6 protein-coding genes. At the same time, linkage disequilibrium (LD) analysis was performed on these regions with significant signals. The results showed that three SNPs in the 55–56 Mbp region of chromosome 3 were highly correlated with the leader SNP (chr3:55526954) that affected Pro content (*r*^*2*^ > 0.6). Similarly, LD analysis showed that there were three SNPs in the 21.2–21.6 Mbp region of chromosome 13, which were highly correlated with leader SNP (chr13:21421661) (*r*^*2*^ > 0.6). Moreover, Through functional enrichment analysis of all candidate genes. The results of GO enrichment analysis showed that several significant GO items were associated with amino acid transport function, including amino acid transmembrane transport and glutamine transport. The results further indicate that these candidate genes are closely associated with amino acid transport. Among them, key candidate genes include *SLC38A1*. For KEGG enrichment analysis, *CACNA2D3* and *CACNA1D* genes were covered by significant pathways.

**Conclusion:**

In this study, GWAS analysis found a total of 28 significant SNPs affecting amino acid content. Through gene annotation, a total of 20 candidate genes were screened. In addition, Through LD analysis and enrichment analysis, we considered that *SERAC1*, *CACNA2D3* and *SLC38A1* genes are important candidate genes affecting amino acid content in duck breast muscle.

**Supplementary Information:**

The online version contains supplementary material available at 10.1186/s12864-024-10287-1.

## Background

With the improvement in living standards, consumers pay more and more attention to the meat quality of livestock and poultry [1]. The nutritional value of protein in meat directly reflects its quality [2]. Amino acids are important components of muscle protein, and the content and composition of amino acids can be used as important indicators for evaluating the nutritional value of meat [3, 4]. The content of essential amino acids in beef is higher than that of other meat products, making beef have high nutritional value [5]. In addition, in pork and mutton studies, different amino acid contents and ratios affect meat quality differently [6, 7]. Asparagine (Asp) and glutamate (Glu) produce umami, and alanine (Ala) and proline (Pro) produce sweetness, which makes meat more delicious. Also, the amino acid content has an extremely important effect on livestock and poultry meat quality.

The amino acid content and proportion in meat are affected by genetics, nutrition, environment, and other aspects [8]. Genetic factors are an important influencing factor [9–11]. Meanwhile, At the beginning of this century, scholars have gradually investigated the underlying genetic mechanism of some chemical compositions in meat. Some studies have revealed quantitative trait loci (QTL) and candidate genes affecting fatty acid content in livestock based on whole-genome scanning and gene linkage analysis with microsatellite markers [12–14]. In recent years, with the rapid development of nucleotide sequencing technology, genome-wide association analysis (GWAS) has been widely used to reveal the genetic foundations of amino acids in meat [15]. Sasago et al. used 22 free amino acids in 574 Japanese black beef as phenotypes for GWAS and The result shows that, the *SLC6A6* gene may affect taurine content, and *STT3B* and *GADL1* genes may affect β-alanine content [16]. In addition, in some bivalves, Meng et al. used the 18 free amino acid contents of 426 oysters as phenotypes for GWAS analysis and identified a total of 787 significant SNPs (*P* < 10^− 6^) [17]. These studies indicate that the GWAS method can be used to study the genetic basis of amino acid content differences in meat.

Duck meat is a high-quality poultry meat widely consumed in a variety of livestock and poultry products, and its rich nutritional value is vital to consumers [18]. Moreover, China is the country with the largest production of duck meat in the world, and the breeding quantity is increasing at an annual rate of 10-15% [19]. Meanwhile, scientists are becoming more concerned about improving the quality of duck meat nowadays. Hence, this study used the GWAS method to locate some QTL regions and candidate genes that can affect amino acid content in duck breast muscle. These findings have important significance for optimizing duck meat production and quality improvement and provide useful references for genetic improvement of livestock and poultry breeding.

## Results

### Descriptive statistics of phenotypic traits

This study used a fully automated amino acid analyzer to determine meat’s amino acid contents, with an amino acid standard of 17 amino acids. Sixteen amino acids were finally detected, including 11 essential amino acids (EAA) and 5 non-essential amino acids (NEAA) (Table 1). However, high concentrations of hydrochloric acid destroyed (Cys)2 during pre-treatment, so it was not detected. In addition, among the 16 amino acids detected, Glu accounted for the highest proportion of total amino acids, and Met accounted for the lowest (Figure S1a). Moreover, among the 11 essential amino acids, the content of Lys and Arg accounted for a relatively high proportion of the total essential amino acids. (Figure S1b) For non-essential amino acids, Glu and Tyr accounted for a relatively high proportion of total non-essential amino acids (Figure S1c).

**Table 1:** Determination results of 16 amino acids in duck breast muscle (g/100 g)

Trait	N	Mean ± S.D.	C.V(%)
EAAs	-	24.05 ± 0.39	-
Thr	344	2.09 ± 0.43	0.21
Gly	344	1.69 ± 0.36	0.21
Val	344	1.85 ± 0.35	0.19
Met	340	1.23 ± 0.17	0.14
Ile	344	1.78 ± 0.34	0.19
Leu	344	3.22 ± 0.48	0.15
Phe	344	1.73 ± 0.31	0.18
His	344	1.77 ± 0.32	0.18
Lys	344	3.72 ± 0.65	0.18
Arg	344	3.53 ± 0.59	0.17
Pro	343	1.44 ± 0.31	0.21
NEAAs	-	15.27 ± 0.51	-
Asp	344	4.00 ± 0.57	0.14
Ser	344	1.76 ± 0.37	0.21
Glu	344	5.61 ± 0.78	0.14
Ala	344	2.31 ± 0.50	0.22
Tyr	342	1.59 ± 0.37	0.23
TAAs	-	39.58 ± 1.09	0.03

*Note*: Asp, Asparagine; Thr, Threonine; Ser, Serine; Glu, Glutamate; Gly, Glycine; Ala, Alanine; Val, Valine; Met, Methionine; Ile, Isoleucine; Leu, Leucine; Tyr, Tyrosine; Phe, Phenylalanine; Lys, Lysine; His, Histidine; Arg, Arginine; Pro, Proline. EAAs are essential amino acids. NEAAs are non-essential amino acids. TAAs are total amino acids. The same is below

**Fig. 1:**
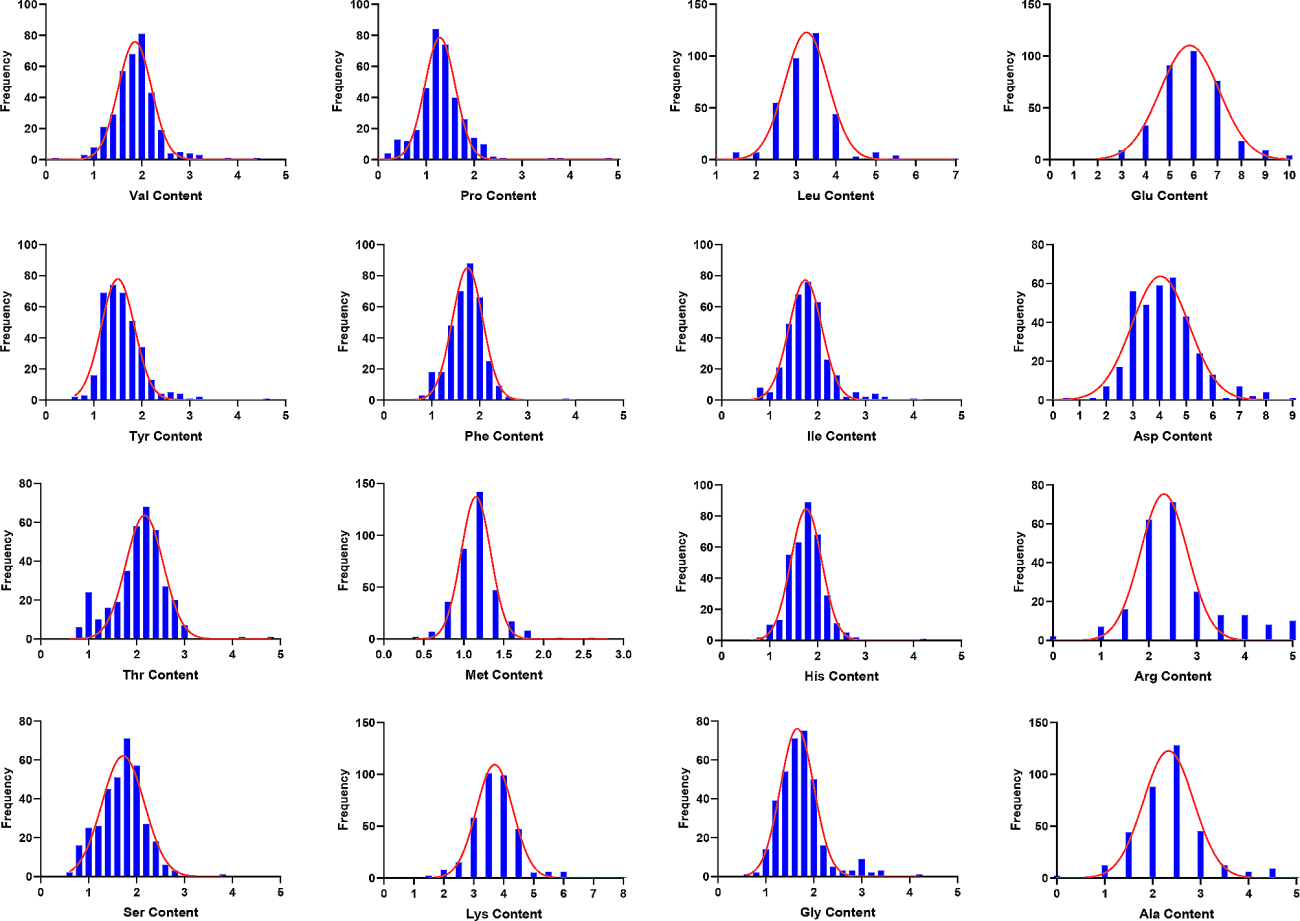
Distribution of frequency of 16 amino acids in duck breast muscle. Asp, Asparagine; Thr, Threonine; Ser, Serine; Glu, Glutamate; Gly, Glycine; Ala, Alanine; Val, Valine; Met, Methionine; Ile, Isoleucine; Leu, Leucine; Tyr, Tyrosine; Phe, Phenylalanine; Lys, Lysine; His, Histidine; Arg, Arginine; Pro, Proline. The same is below

**Fig. 2:**
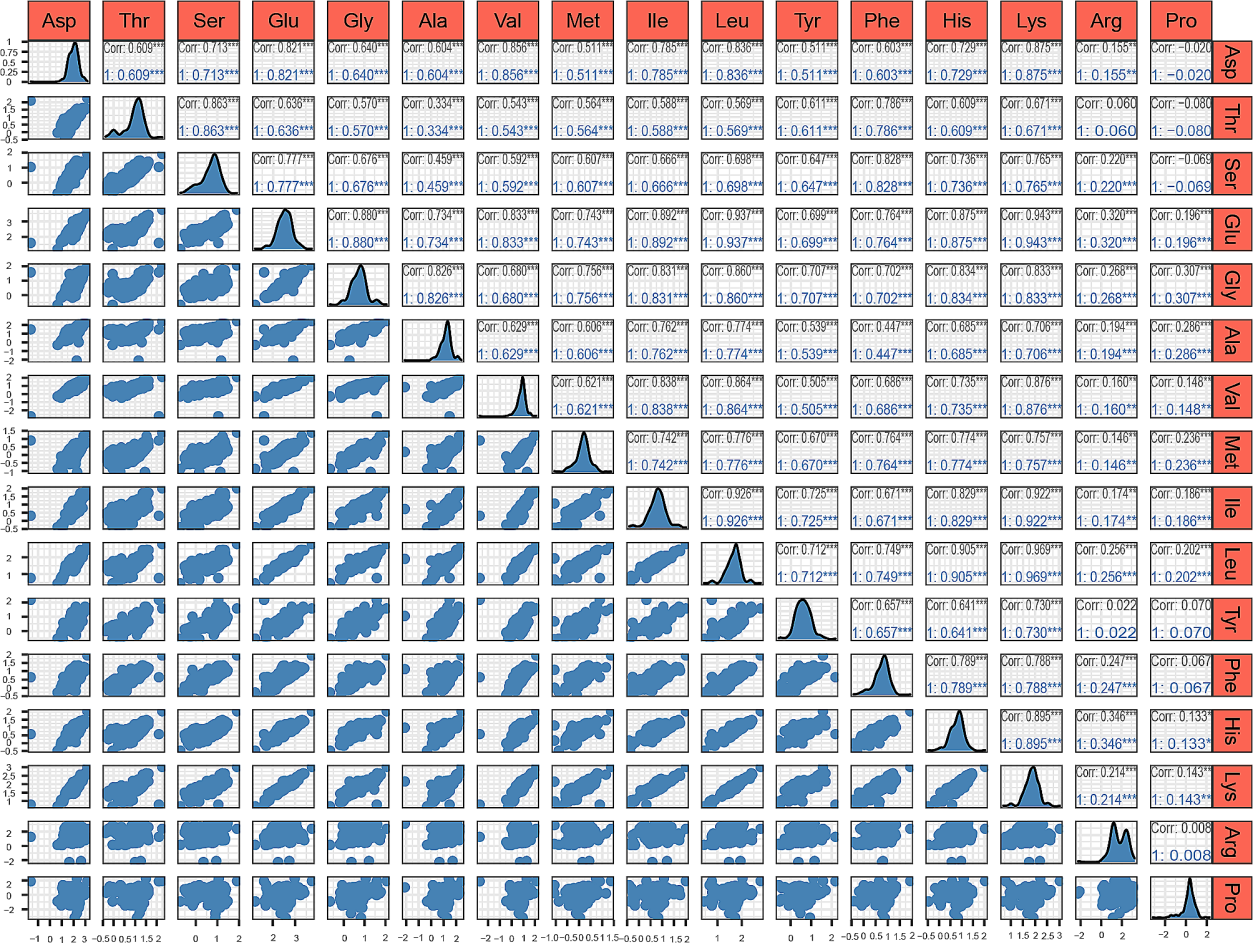
The correlation coefficient matrix heat map of 16 amino acids. Pearson’s correlation coefficients and p-values were calculated using the cor. Test function in the R stats package. *p < 0.05 was considered statistically significant; **p < 0.01 was considered very significant; and ***p < 0.001 was considered highly significant

Moreover, by comparative analysis, the contents of 3 amino acids showed an apparent difference between the genders (*P* < 0.05), including Ser, Met, and Phe (Table S1). Furthermore, the normal distribution test shows that the 16 amino acid content values were normally distributed (Fig. 1). In addition, we performed principal component analysis (PCA) on the content of 16 amino acids in all individuals. The results showed that amino acid content did not show significant population stratification (Figure S2).In addition, through correlation analysis, except for Arg and Pro, which had low correlation with other amino acids, the correlation among the other 14 amino acids was relatively high, with Pearson correlation coefficients ranging from 0.33 to 0.96 (Fig. 2). Among them, Val, Gly, Ala, Glu, and Tyr were highly correlated, and their Pearson correlation coefficients ranged from 0.50 to 0.89.

### Genome-wide association analysis

In this study, GWAS analysis was conducted using the absolute and relative contents of 16 amino acids in the breast muscles of 120-day-old ducks as phenotypes (Correction threshold = 8.59). The absolute content represents the actual value of the amino acid we measured, and the relative content is equal to the absolute content divided by the total amino acid content. Their meanings are different. At the same time, relative content can be used as a phenotype to verify the GWAS results of absolute content. Significant SNP signals were observed for Pro, Tyr, Val, and Glu in the Manhattan plot when using absolute content as the phenotype. Additionally, using the relative content as the phenotype, the GWAS results showed significant SNP signals for Tyr, Pro, Ala, Val, and Gly. Furthermore, the corresponding Q-Q plots demonstrated that the analysis model used was reasonable, with most p-values consistent with expected values and significant SNPs found, demonstrating the reliability of the above association results. Manhattan plots and Q-Q plots for other traits that showed no significant signals can be found in Figure S3 and S4, respectively.

### GWAS based on absolute content

**Fig. 3:**
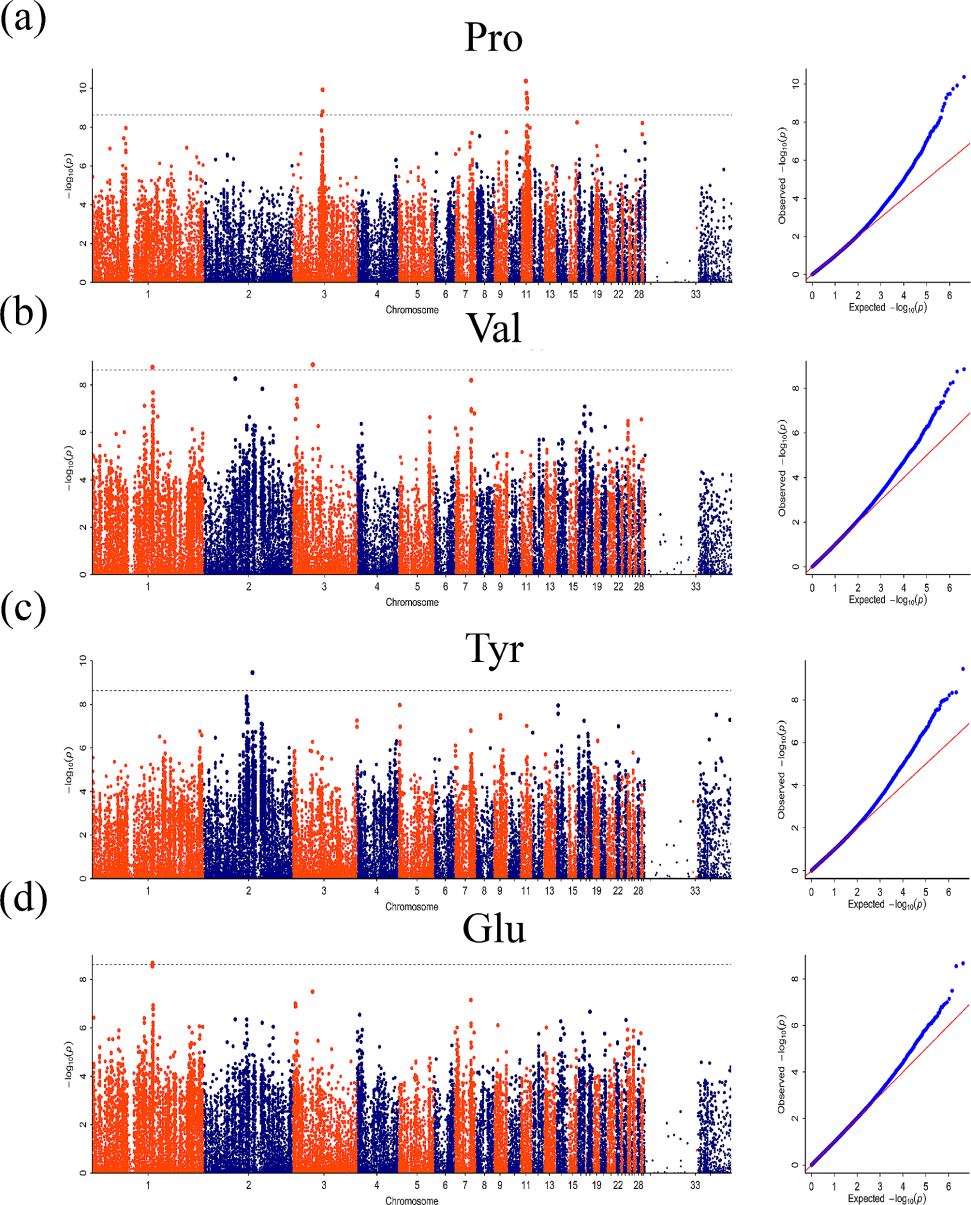
Manhattan plots of genome-wide association analysis results. The absolute contents of amino acids as phenotypes were analyzed by GWAS to obtain the Manhattan map. Pro = Proline; Val = Valine; Tyr = Tyrosine; Glu = Glutamate. The x-axis shows the physical positions of each marker along the chromosomes, and the y-axis shows the − Log_10_*P* values for the association tests. The dashed line represents the threshold line (Correction threshold = 8.59)

The absolute content of amino acids was used as a phenotype for GWAS analysis, and several characters with significant signals were obtained. Among them, the Manhattan map of Pro has significant SNP signals on chromosomes 3 and 11 (Fig. 3a). A total of 9 SNPs reached the significant threshold level, including three on chromosome 3 and 6 on chromosome 11. Through functional gene annotation of SNPs, *SERAC1*, *NPTN*, *ZNF592*, and *SH3GL3* were screened (Table 2). GWAS results from Val showed only two SNPs that reached the significant threshold level (Fig. 3b), and these SNP-annotated genes include *TTC7A* and *LOC110351546* (Table 2). The Manhattan result of Tyr showed a significant SNP signal on chromosome 2 (Fig. 3c), which was distributed on *CDH18* (Table 2). The results of Glu showed a significant SNP signal on chromosome 1 (Fig. 3d), within the region of the *LOC110351546* gene (Table 2).

**Table 2:** Annotation of functional genes for significant SNPs

Trait	POS	REF	ALT	SNPs-pos on gene	P-value	Candidate Gene
Pro	chr3:53416575	G	A	Intergenic region	8.615655883	-
	chr3:55526847	A	G	Upstream gene	8.806510401	SERAC1
	chr3:55526954	G	A	Upstream gene	9.925927754	SERAC1
	chr11:10098877	A	G	Intron	10.37985867	NPTN
	chr11:11473105	A	G	Intron	9.752220789	ZNF592
	chr11:12657409	A	G	Intron	9.457450333	SH3GL3
	chr11:12657416	A	G	Intron	9.483578608	SH3GL3
	chr11:12657904	C	T	Intron	8.973790052	SH3GL3
	chr11:13126364	T	C	Intron	9.268837058	LOC110354051
Val	chr1:111670282	A	G	Intron	8.752708159	LOC110351546
	chr3:37135312	A	G	Intron	8.858523538	TTC7A
Tyr	chr2:90454652	T	G	Intron	9.457222813	CDH18
Glu	chr1:111670282	A	G	Intron	8.673653256	LOC110351546

*Note*: These significant traits were obtained by GWAS analysis of the absolute contents of 16 amino acids as phenotypes. Pro, Proline; Val, Valine; Tyr, Tyrosine; Glu, Glutamate

### GWAS based on relative content

**Fig. 4:**
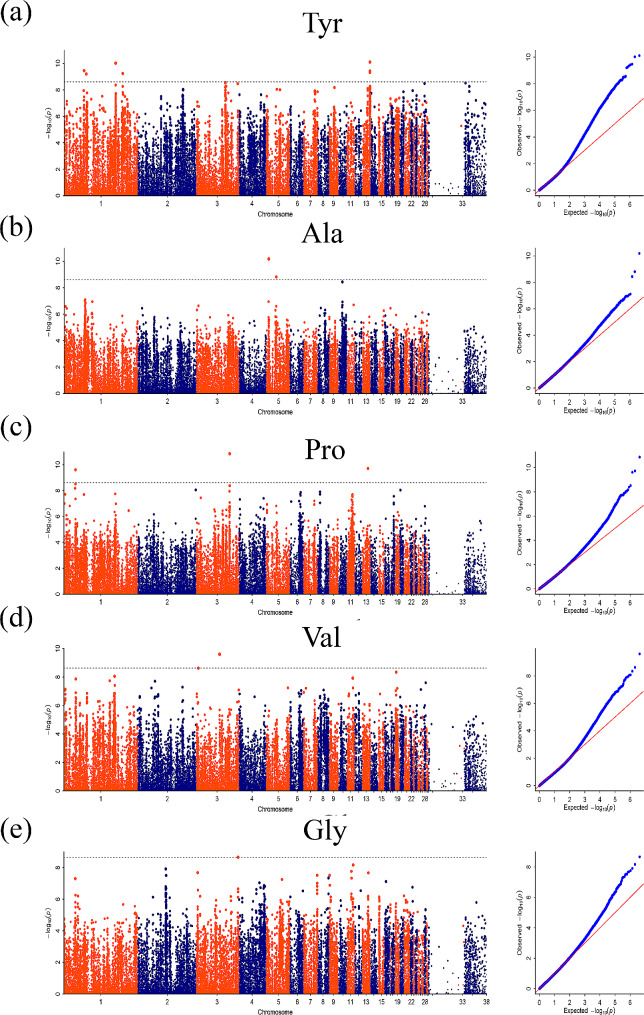
Manhattan plots of genome-wide association analysis results. The relative content of amino acids was used as the phenotype for GWAS analysis to obtain the Manhattan map. Tyr = Tyrosine; Ala = Alanine; Pro = Proline; Val = Valine; Gly = Glycine. The x-axis shows the physical positions of each marker along the chromosomes, and the y-axis shows the − Log_10_*P* values for the association tests. The dashed line represents the threshold line (Correction threshold = 8.59)

Similarly, GWAS analysis using amino acid relative content as phenotype also obtained some characters with significant signals. The Manhattan result of Tyr showed 7 significant SNPs (Fig. 4a), of which four were located on chromosome 1 and three on chromosome 13. The functional gene annotations of SNPS are shown in Table 3, including *SYN3*, *BICD1*, *MYO16*, and *CACNA1D* and *CACNA2D3*. The Manhattan result of Ala showed significant SNP signals on chromosome 5 (Fig. 4b). The *SYT16* and *TNNI2* genes were obtained by SNP annotation (Table 3). The Manhattan map of Pro showed only three significant SNP signals (Fig. 4c), and their SNP-annotated genes include *SLC38A1* (Table 3). In addition, Val’s GWAS results showed significant SNP signals on chromosome 3 (Fig. 4d), and *PAK5* and *HSF2* were screened out through SNP annotation (Table 3). The Manhattan map of Gly showed a significant SNP signal on chromosome 3 (Fig. 4e), which was annotated to acquire the *INTS9* gene (Table 3).

**Table 3:** Annotation of functional genes for significant SNPs

Trait	POS	REF	ALT	SNPs-pos on gene	P-value	Candidate Gene
Tyr	chr1:56024262	A	G	Intron	9.464962341	SYN3
	chr1:62379647	T	C	Intron	9.209577756	BICD1
	chr1:145129100	T	C	Upstream gene	10.03190177	MYO16
	chr1:164861312	A	G	Intergenic region	9.249332864	-
	chr13:21170995	T	C	Intron	9.425972941	CACNA1D
	chr13:21293369	A	C	Intergenic region	9.33571271	-
	chr13:21421661	C	G	Intron	10.11347236	CACNA2D3
Ala	chr5:7072617	A	G	5’prime-UTR	10.18759205	SYT16
	chr5: 28,476,864	C	T	Intergenic region	8.815715825	TNNI2
Pro	chr1:32710401	C	T	Intron	9.606168973	SLC38A1
	chr3:93598214	T	C	Intergenic region	10.86480489	-
	chr13:15682406	T	C	Upstream gene	9.709181737	LOC113845051
Val	chr3:5990965	T	C	Intron	8.626157739	PAK5
	chr3:65610946	T	C	Intron	9.607422093	HSF2
Gly	chr3:117723253	C	T	Intron	8.650504302	INTS9

*Note*: These significant traits were obtained by GWAS analysis of the relative contents of 16 amino acids as phenotypes. Relative content value equals absolute content value divided by total amino acid value. Tyr, Tyrosine; Ala, Alanine; Pro, Proline; Val, Valine; Gly, Glycine

### LD analysis of significant candidate regions

**Fig. 5:**
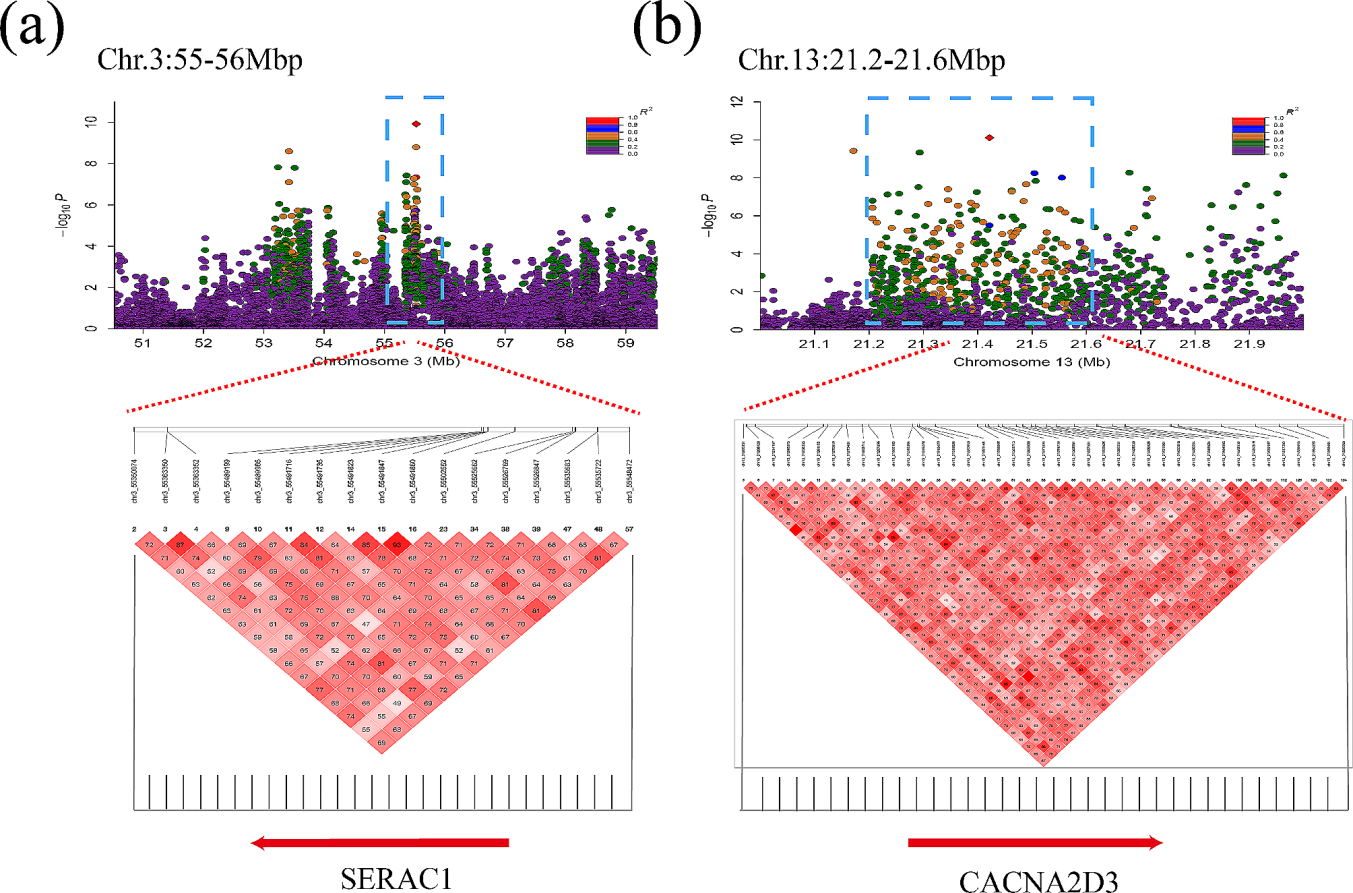
LD analysis in the region with significant SNPs. (**A**) represents the LD analysis and related genes in the c significant SNPs region (the Manhattan map showed a significant signal after GWAS phenotypic with the absolute content of Pro). (**B**) represents LD analysis and related genes in the significant SNPs region of Tyr (Manhattan map showed significant signal after GWAS with the relative content of Tyr as the phenotype)

According to the GWAS results, Pro had significant candidate regions on chromosomes 3 and 11(Fig. 3a). In this region (55.35-55.54Mbp), the correlation between leader SNP (chr3:55526954) and the surrounding SNPs was obtained through LD analysis, and 3 SNPs were highly correlated (*r*^*2*^ > 0.6; Fig. 5a). Finally, we screened the *SERAC1* gene (Table S2). Similarly, Tyr has significant SNPs on chromosome 13 (Fig. 4a). Through LD analysis, the correlation between leader SNP (chr13:21421661) and other SNPs was calculated in this region (21.2-21.6Mbp), of which 3 SNPs were highly correlated (*r*^*2*^ > 0.6; Fig. 5b). Finally, the *CACNA2D3* gene was screened by gene annotation (Table S3).

### Functional enrichment analysis of candidate genes located in SNPs

**Fig. 6:**
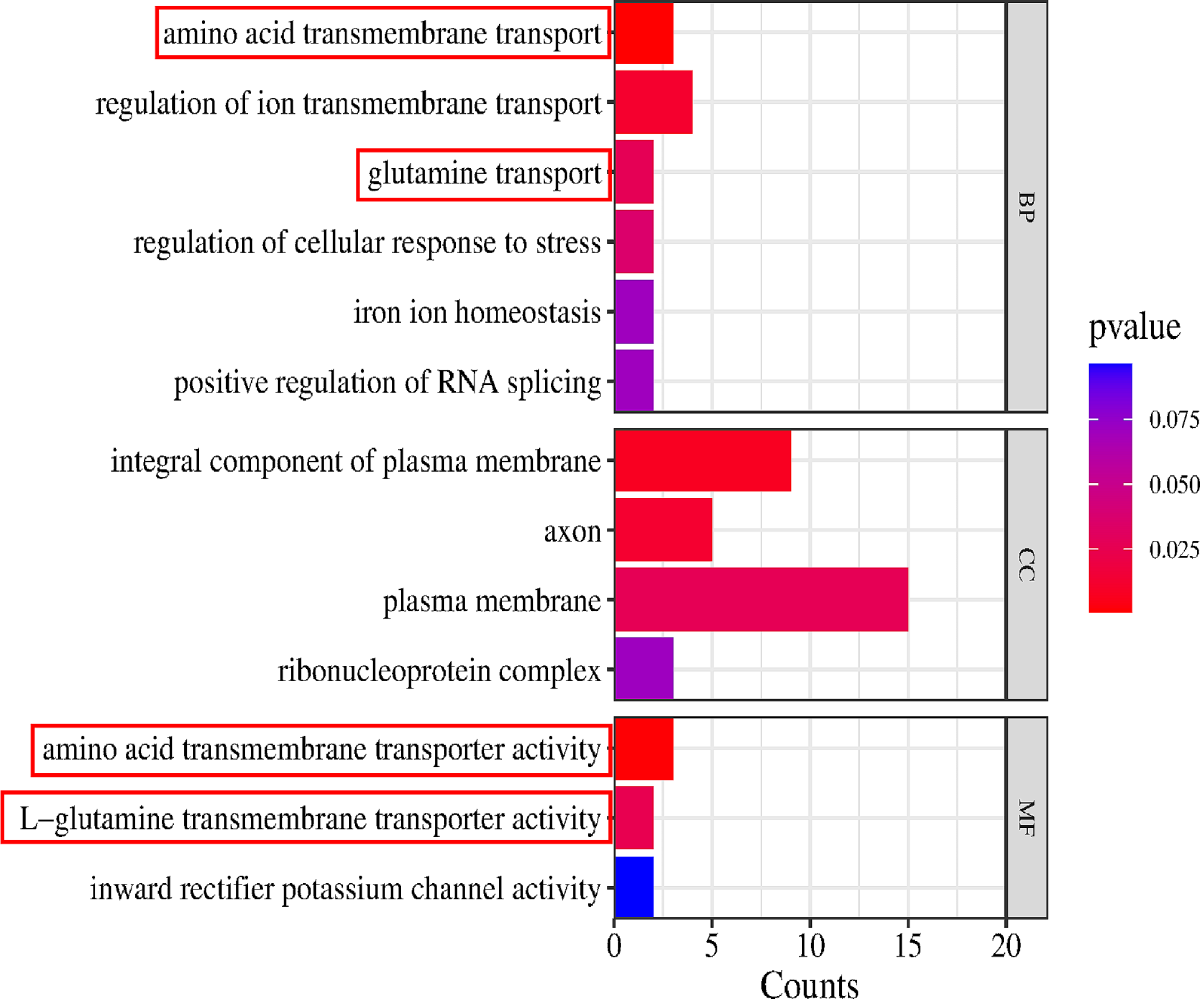
GO enrichment analysis for 134 candidate genes. The x-axis indicates the number of genes for each GO term; the y-axis corresponds to the GO terms. The color of the bar represents the P value. The red boxes represent the GO terms associated with amino acids

To further investigate the functional and regulatory relationships of these significant SNP markers and their various candidate genes, we used GO and KEGG databases for functional enrichment analysis of 134 genes, including all the annotated genes of significant SNPs and the significant locus near the high-LD region (*r*^*2*^ > 0.2) of annotated genes (Table S4). GO enrichment results showed amino acid transmembrane transport as a significant GO term in the biological process. Similarly, glutamine transport was also significant. There are 3 GO subcategories in the molecular function, two associated with amino acid transport and transport function, including amino acid transmembrane transporter activity and L-glutamine transmembrane transporter activity. Among them, the important candidate genes associated with amino acid transport function include *SLC38A1* and *SLC38A2* genes (Fig. 6, Table S5). For KEGG enrichment analysis, only one significant pathway (Cardiac muscle contraction pathway) was enriched (Table S6). Genes enriched in this pathway include *CACNA2D3*, *CACNA1D* and *CACNA2D4*.

## Discussion

Amino acids are the raw materials of protein in poultry meat. At the same time, the amino acid content is an important index of meat quality [20]. This study detected the content of 16 amino acids in the breast muscles of 358 ducks. The content of Glu (5.61 g/100 g) and Asp (4.00 g/100 g) is relatively high, and the content of Met (1.23 g/100 g) is relatively low. Studies have shown that Glu and Asp have relatively high amino acid content in duck meat [21, 22], which is consistent with our results. However, the content values of some amino acids obtained in this study differ greatly from the results of other studies. Amino acids such as glutamate and asparagine acid content values were 2 times higher than those of other studies [23]. This may be related to many factors, among which we think it is closely associated with the age factor, because our slaughter age is 120 days old, and other studies slaughter time and our slaughter time gap is larger. At the same time, Studies have shown that within a certain range, the older the poultry animals are, the higher the amino acid content will be [24]. Of course, it is closely associated with the breed, sex, and nutritional factors. In addition, compared with other livestock and poultry meat, the contents of various amino acids are also different [25]. Zagorska et al. showed that the content of Glu, Lys, Asp, and Leu in pork was relatively high, and its content was between 6% and 13% of total amino acids (dry weight) [26]. This is quite different from our results. This may be due to the differences in the amino acid content of duck meat and other poultry meat due to genetic background, nutrition, and feeding methods [27].

Moreover, we also compared and analyzed the differences in these amino acids between males and females and found that only 3 amino acids had significant differences between males and females (*P* < 0.05), including Ser, Met, and Phe. In previous studies, Yin et al. showed that the 11 amino acids of Guangyuan grey chickens showed significant differences between genders [28]. Ji et al. also compared the amino acid content in different parts of Duroc pigs and found significant differences in various amino acids between genders [29]. Although the finding is inconsistent with our results, it is also a normal phenomenon due to the difference in breed, nutrition, and other factors.

Phenotypic correlation analysis showed that most amino acids had a high correlation, such as Lys and Leu, Tyr and Phe. In the study of amino acid metabolism, the results show that the metabolism of most amino acids is interrelated [30]. For example, Tyr can be synthesized from Phe, and the synthesis of Pro requires the participation of Glu [31–33]. However, the specific influencing mechanism needs further systematic study.

Based on GWAS analysis of different phenotypic values, significant SNPs were found in Pro, Tyr, and other traits. We found candidate genes affecting Pro content on chromosomes 3 and 11 through gene annotation, including *SLC38A1*, *SERAC1*, *ZNF592*, *SH3GL3*, and *NPTN*. According to reports, the *SLC38A1* belongs to the *SLC38A* family. It is involved in the transport of Ala and Gln in mammals and participates in the TCA cycle [34, 35]. On the other hand, studies have reported that Gln is involved in Pro metabolism [36]. Therefore, we speculate that the *SLC38A1* gene is closely associated with the metabolic process of Pro. In addition, studies have shown that Pro’s metabolic process occurs in mitochondria [37]. Notably, *SERAC1* plays a key role in phosphatidylglycerol remodeling, which is essential for both mitochondrial function and intracellular cholesterol transport [38]. However, whether this is relevant for pro-synthesis in mitochondria needs further study. Similarly, we screened candidate genes that affect Tyr content, including *CACNA2D3*, *CDH18*, *SYN3*, *BICD1*, *MYO16*, and *CACNA1D*. Interestingly, Yang et al. presented candidate genes affecting Australian Boer goat muscle development through GWAS analysis, including the *CACNA2D3* gene [39]. As an important amino acid constituting muscle protein [40], it is speculated that this may be indirectly associated with the metabolic process of tyrosine. However, whether there is a link between the two needs to be further studied.

Through GO and KEGG analyses, Significant GO items of candidate gene enrichment were associated with amino acid transmembrane transport activity. Interestingly, both SLC38A1 and SLC38A2 genes are included in the GO terms associated with amino acid transport. Therefore, we speculate that these candidate genes may be important in material transport during amino acid production. For KEGG analysis, only one significant pathway was enriched in this study, which included *CACNA2D3* and *CACNA1D*. However, whether this pathway is involved in amino acid regulation needs further study.

Amino acids have an important effect on meat quality. However, the genetic mapping of amino acid-related genes in poultry has not been reported. Therefore, the candidate genes affecting amino acid content in duck breast muscle were identified by GWAS for the first time in this study. Due to the extremely complex amino acid gene regulation and metabolism process, small sample size, and differences in detection methods, the substantive evidence we finally obtained is still limited. However, these candidate genes and candidate regions can provide a useful reference value for breeding breeds with better meat quality by genetic means.

## Materials and methods

### Animals and sampling

A total of 358 ducks (Tianfu Nonghua duck) were provided by the Waterfowl Breeding Farm of Sichuan Agricultural University. All ducks were raised under the same conditions and had free access to food and water. At 60 days old, 5 mL of whole blood samples were collected using venipuncture and stored at -20 ℃. At 120 days old, breast muscles were collected during slaughter and stored in a -4℃ refrigerator. The raising of animals, and sampling processes were achieved through the guidelines of the Sichuan Agricultural University institution and met the feeding standards of the Animal Welfare Committee.

### Determination of amino acid content

Amino acid content was detected in the breast muscles of 358 ducks at 120 days of age. The detection process was divided into pre-processing and on-machine operation.

The duck breast muscle was pre-treated by acid hydrolysis. First, an accurate weight of 0.5 g duck breast muscle sample collected from the same location was carefully added into the hydrolytic tube. Addition of 6 mol/L HCL10 mL containing phenol was made, sealed, and baked in an oven at 110℃ for 24 h. Secondly, the volume was set to 100 mL after the sample filtration with ultra-pure water. Third, ultrafiltration was carried out using a C_18_ extractor. Fourthly, after ultrafiltration, the sample solution was absorbed into a 1.5mL EP tube and deacidified in a vacuum deacidifier (about 2–3 h). Finally, 2mL of sample buffer was added to the deacidified sample and mixed evenly. Then, a small amount of liquid was absorbed by a needle tube and filtered through a 0.25 μm filter into a small brown bottle ready for machine analysis.

In addition, 17 amino acid standards were used (Sigma, AAS18). After pretreatment, amino acids were determined by an automatic amino acid analyzer (A300, membraPure GmbH, Germany). For specific operation methods and processes refer to the manual (Version 1.3 of the A300). The peak of amino acid content is shown in Figure S5.

### DNA extraction and whole genome re-sequencing

DNA was extracted from the duck’s blood (*n* = 358) using the phenol-chloroform protocol. DNA quality was detected by Nano Drop-2000 and agarose gel electrophoresis. The evaluated samples were constructed by a paired-end library. A small fragment library with a fragment length of 150 base pairs (bp) (PE150) was constructed, and a successful library was constructed. All libraries were sequenced on the Illumina®Hiseq X-Ten platform of Bio-company (Biomarker Technologies, Beijing, China). Each sample’s mean sequence coverage of the raw reads sequence was 5×.

Quality control analysis of raw reads was performed using Trimmomatic (v0.36) after removing reads containing joint sequences. The clean reads obtained were then compared with the duck reference genome (ZJU1.0, GCA_015476344.1) using Burrows-Wheeler Aligner (BWA aln) [41]. Meanwhile, a total of 12,996,047,606 clean reads were generated after quality filtering. HaplotypeCaller in GATK was used to identify and analyze SNPs and InDels, and VCFtools was used to filter SNP data further [42, 43]. Finally, a total of 19,628,941 SNPs were obtained by VCFtools for GWAS analysis. The specific methods and statistical results have been sorted out by our research group in the early stage [44].

### Genome-wide association analysis (GWAS)

GWAS uses the mixed linear model program Emmax to identify SNPS [45]. To correct for population stratification, the model uses fixed effects that include the first three principal component values (PCA eigenvectors) from the genome-wide SNP genotypes [46]. Meanwhile, random effects are present in the kinship estimation matrix of all individual genome-wide SNP genotypes. In addition, the effect of sex was further analyzed as a fixed effect in GWAS. The R package (v3.5.1) was used to draw Manhattan and QQ plots. Finally, the Correction threshold (− Log_10_*P* ≥ 8.59) was used to identify significant SNPs, and the calculation formula is *P* = 0.05/The total number of SNPs. Meanwhile, the QQ plots are used to detect if SNP are false positives due to population stratification.

### LD analysis

After GWAS analysis, we screened some important candidate regions and SNPs. To explore the relationship between the most significant SNPs (the leader SNPs) and other SNPs in the significant candidate region, this study used Plink (version 1.90) software for LD analysis, and the Locuszoom graph was generated by R (version 3.5.1).

### Gene annotation and enrichment analysis

Based on the duck reference genome (ZJU1.0, GCA_015476345.1), SnpEff software annotated significant SNPs to obtain candidate genes for different traits [47]. David website (https://david.ncifcrf.gov/) was used for Gene Ontology (GO) function enrichment, and KOBAS 3.0 (http://kobas.cbi.pku.edu.cn/) was used for the Kyoto Encyclopedia of Genes and Genomes (KEGG) pathway enrichment analysis. In the enrichment analysis, a significant level of *P* < 0.05 was the standard for significant enrichment of GO terms and KEGG pathways.

### Statistical analysis

Microsoft Excel 2021 software was used to analyze each trait’s mean value, standard deviation, and coefficient of variation. SPSS software (version 22.0, Windows, SPSS Inc., Chicago, IL) was used for the normal distribution test. The data that did not conform to the normal distribution was processed by logarithm, and Rdrew the correlation analysis and correlation heat map between various traits. The graphs were drawn with GraphPad Prism (version 8.0.2) and R Studio (version 4.1.1).

### Electronic supplementary material

Below is the link to the electronic supplementary material.

Supplementary Material 1

Supplementary Material 2

Supplementary Material 3

## Data Availability

The genome re-sequencing raw data was available in NCBI’s SRA database (https://trace.ncbi.nlm.nih.gov/Traces/sra/sra.cgi?view=studies&f=study&term= &go = Go; Accession number: PRJNA907492 and PRJNA907501).

## Abbreviations

GWAS: Genome-wide association studies
QTLs: Quantitative trait loci
LD: Linkage disequilibrium
SNP: Single nucleotide polymorphism
PCA: Principal component analysis
KEGG: Kyoto Encyclopedia of Genes and Genomes
GO: Gene ontology
Asp: Asparagine
Thr: Threonine
Ser: Serine
Glu: Glutamate
Gly: Glycine
Ala: Alanine
Val: Valine
Met: Methionine
Ile: Isoleucine
Leu: Leucine
Tyr: Tyrosine
Phe: Phenylalanine
Lys: Lysine
His: Histidine
Arg: Arginine
Pro: Proline
EAAs: essential amino acids
NEAAs: non-essential amino acids
TAAs: total amino acids
