# Supplementary material for: Genome-wide association analysis explores the genetic loci of amino acid content in duck’s breast muscle
Source: BMC Genomics. 2024 May 16;25:486. doi: 10.1186/s12864-024-10287-1 (PMC11097541; doi:10.1186/s12864-024-10287-1)
Supplement: Supplementary file 1 — Supplementary Material 1 [file 12864_2024_10287_MOESM1_ESM.docx]

# Genome-wide association analysis explores the genetic loci of amino acid content in duck’s breast muscle

Rui Wang^1, 2, 3^, Yinjuan Lu^1, 2, 3^, Jingjing Qi^1, 2, 3^, Yang Xi^1, 2, 3^, Zhenyang Shen^1, 2, 3^, Grace Twumasi^1, 2, 3^, Lili Bai^1, 2, 3^, Jiwei Hu^1, 2, 3^, Jiwen Wang^1, 2, 3^, Liang Li^1, 2, 3^, Hehe Liu^1, 2, 3,^ ^*^

^1^Farm Animal Genetic Resources Exploration and Innovation Key Laboratory of Sichuan Province, Sichuan Agricultural University, Chengdu, Sichuan, P.R. China

^2^Key Laboratory of Livestock and Poultry Multi-omics, Ministry of Agriculture and Rural Affairs, Wenjiang District 611130, Chengdu, Sichuan, P.R. China

^3^National Key Laboratory for Swine and Poultry Breeding

*Corresponding Author: Professor Hehe Liu, E-mail: [Liuee1985@sicau.edu.cn](mailto:Liuee1985@sicau.edu.cn)

**Supplementary Figures**


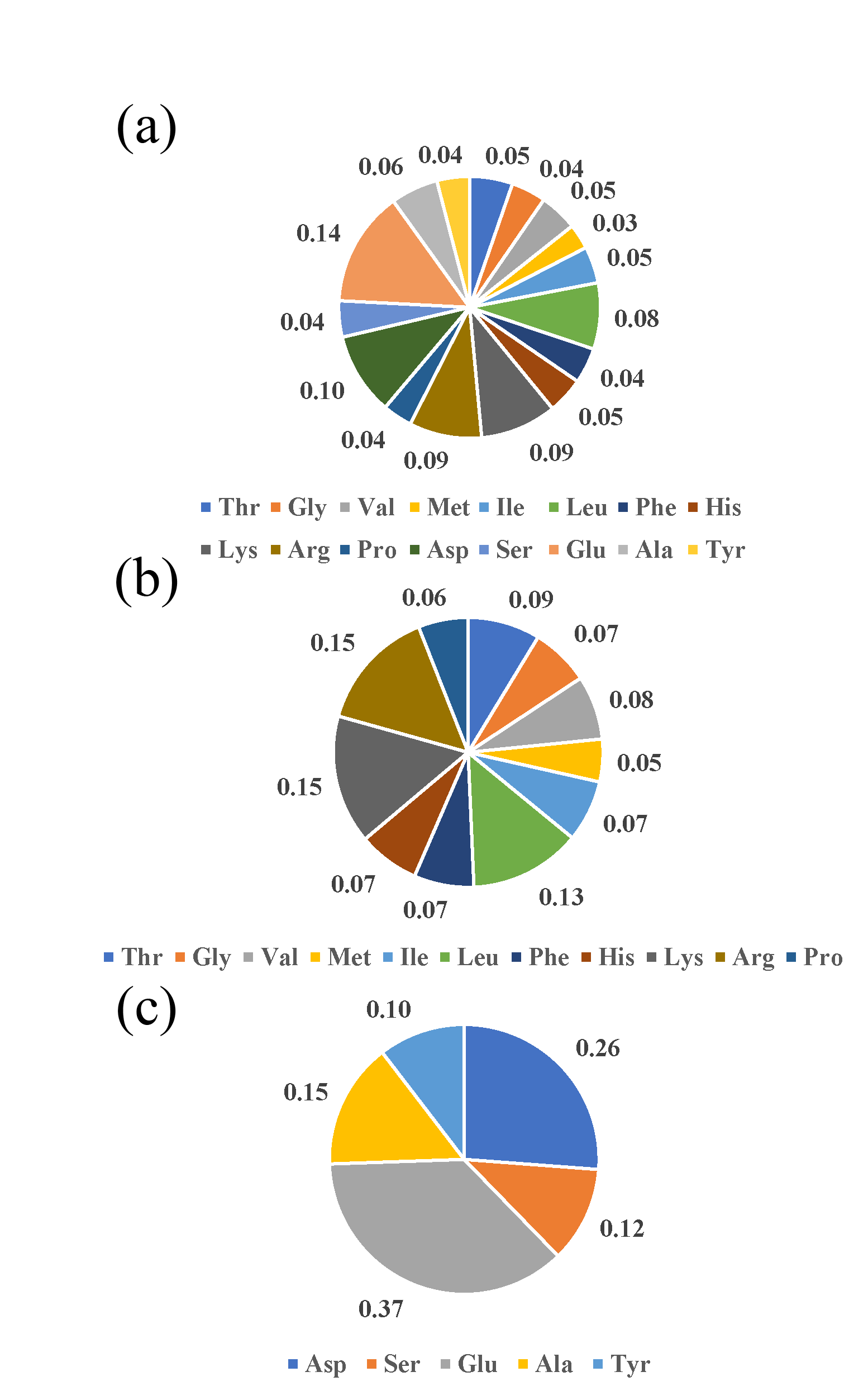


**Figure S1 Amino acid content ratio chart.** (a) represents the proportion of 16 amino acids in total amino acids (%). (b) represents the Proportion of 11 essential amino acids in total essential amino acids (%). (c) represents the Proportion of 5 non-essential amino acids in total non-essential amino acids (%). Asp, Asparagine; Thr, Threonine; Ser, Serine; Glu, Glutamate; Gly, Glycine; Ala, Alanine; Val, Valine; Met, Methionine; Ile, Isoleucine; Leu, Leucine; Tyr, Tyrosine; Phe, Phenylalanine; Lys, Lysine; His, Histidine; Arg, Arginine; Pro, Proline. The same is below.


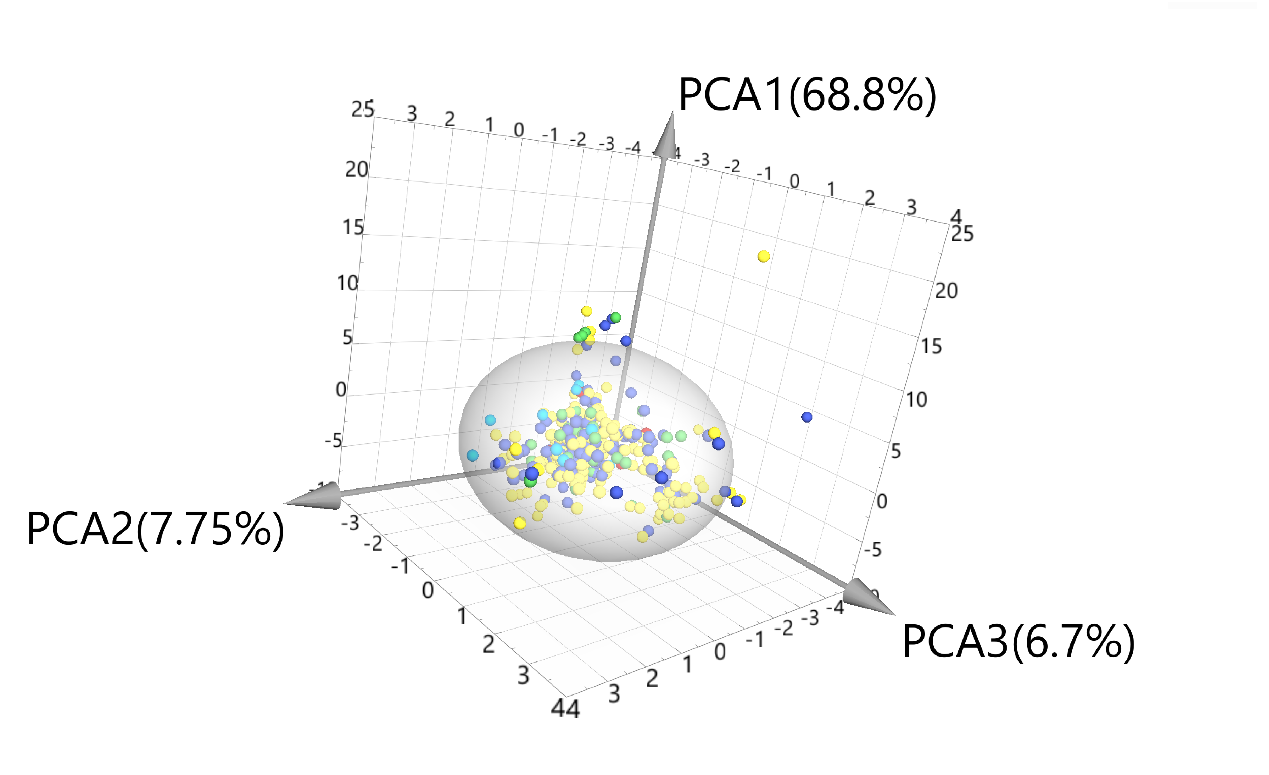


**Figure S2 Principal Component Analysis.** The contents of 16 amino acids in all individuals were analyzed by three-dimensional principal component analysis.


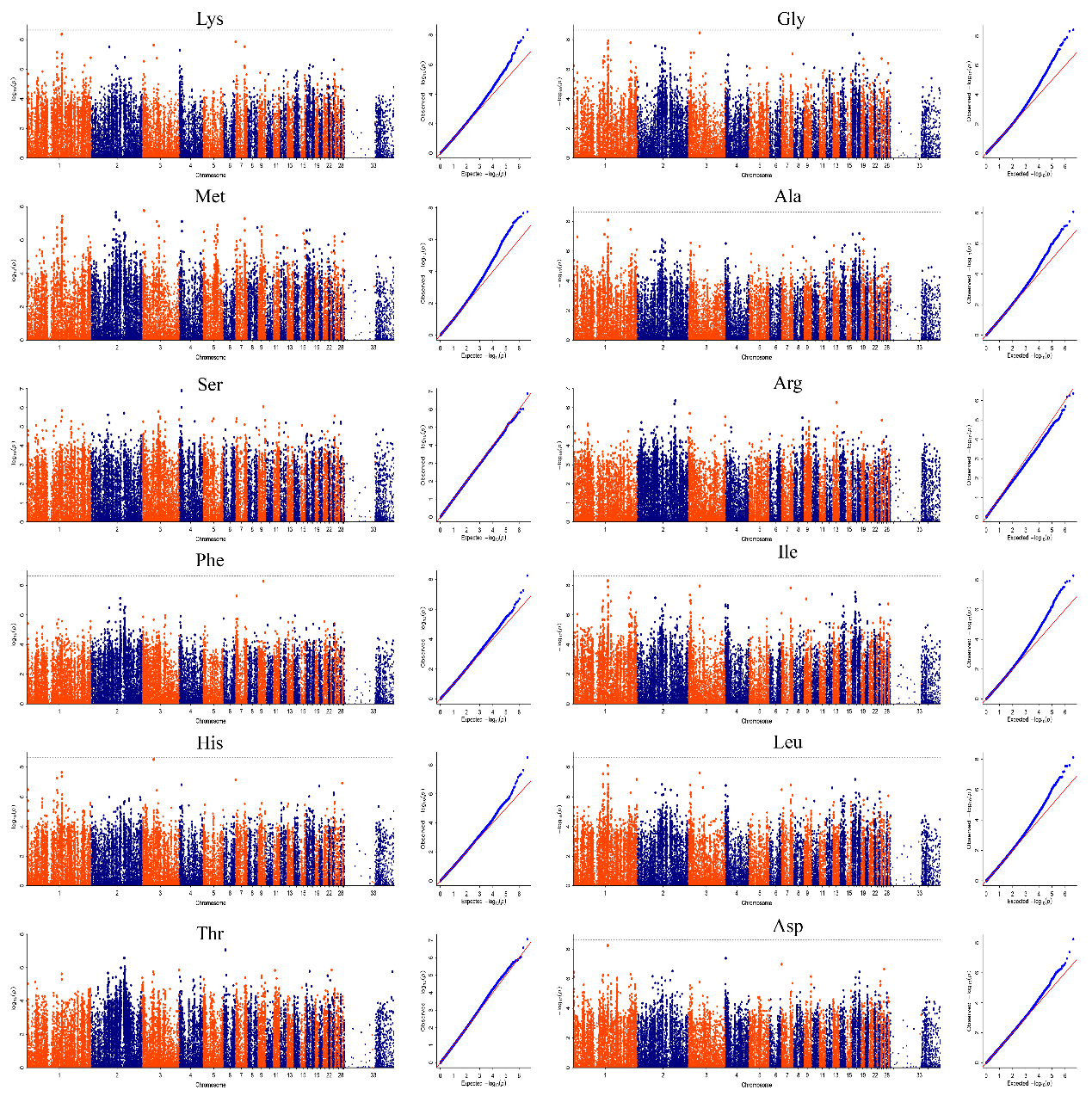


**Figure S3 Manhattan plots of genome-wide association analysis results.** This resulted from GWAS analysis based on absolute amino acid content as phenotype. There was no significant SNP signal. Asp, Asparagine; Thr, Threonine; Ser, Serine; Gly, Glycine; Ala, Alanine; Met, Methionine; Ile, Isoleucine; Leu, Leucine; Phe, Phenylalanine; Lys, Lysine; His, Histidine; Arg, Arginine. The x-axis shows the physical positions of each marker along the chromosomes, and the y-axis shows the −log10 P values for the association tests. The dashed line represents the threshold line (Correction threshold = 8.59).


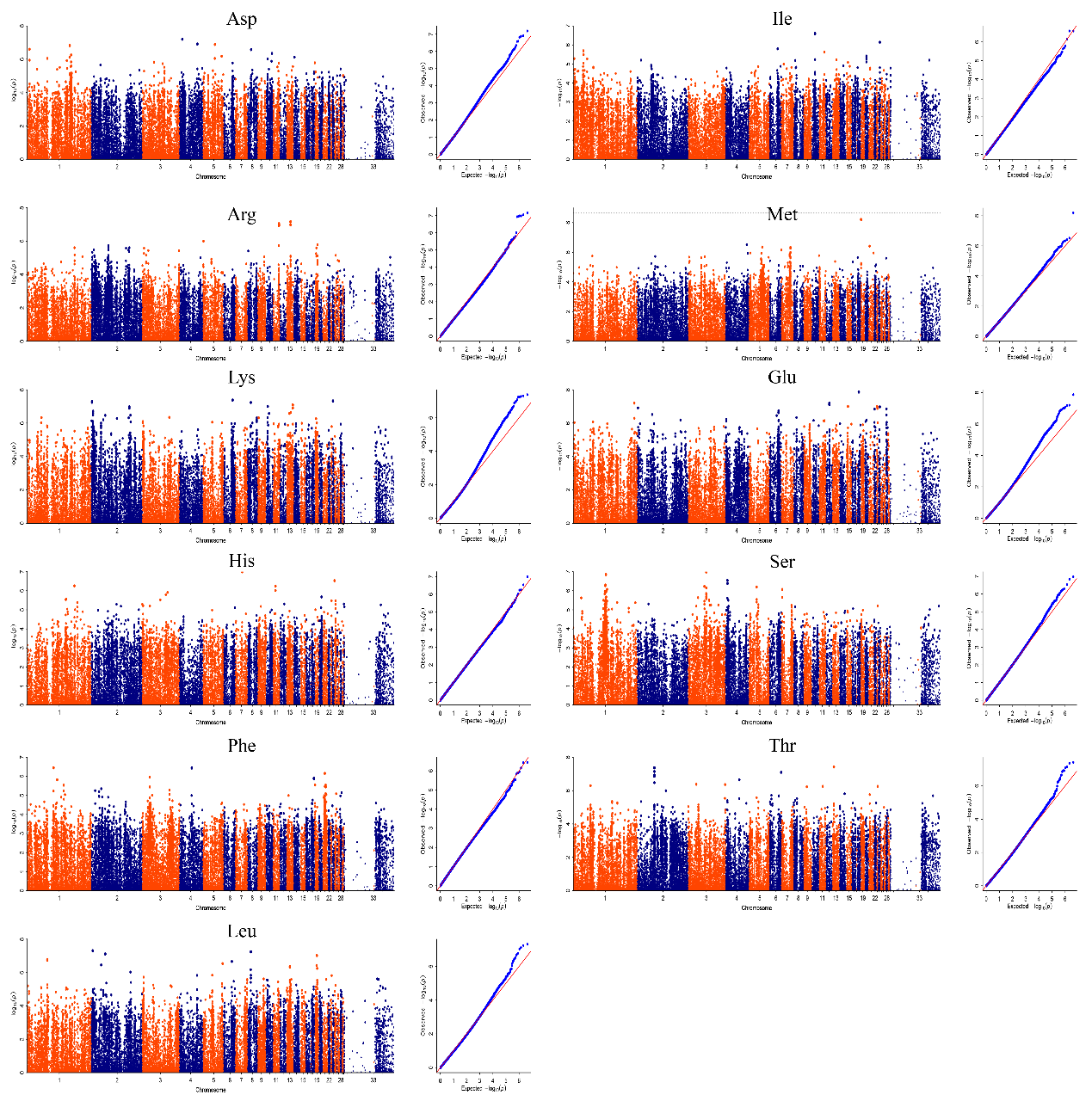


**Figure S4 Manhattan plots of genome-wide association analysis results.** This resulted from GWAS analysis based on amino acid relative content as phenotype. There was no significant SNP signal. Asp, Asparagine; Thr, Threonine; Ser, Serine; Glu, Glutamate; Met, Methionine; Ile, Isoleucine; Leu, Leucine; Phe, Phenylalanine; Lys, Lysine; His, Histidine; Arg, Arginine. The x-axis shows the physical positions of each marker along the chromosomes, and the y-axis shows the −log10 P values for the association tests. The dashed line represents the threshold line (Correction threshold = 8.59).

**
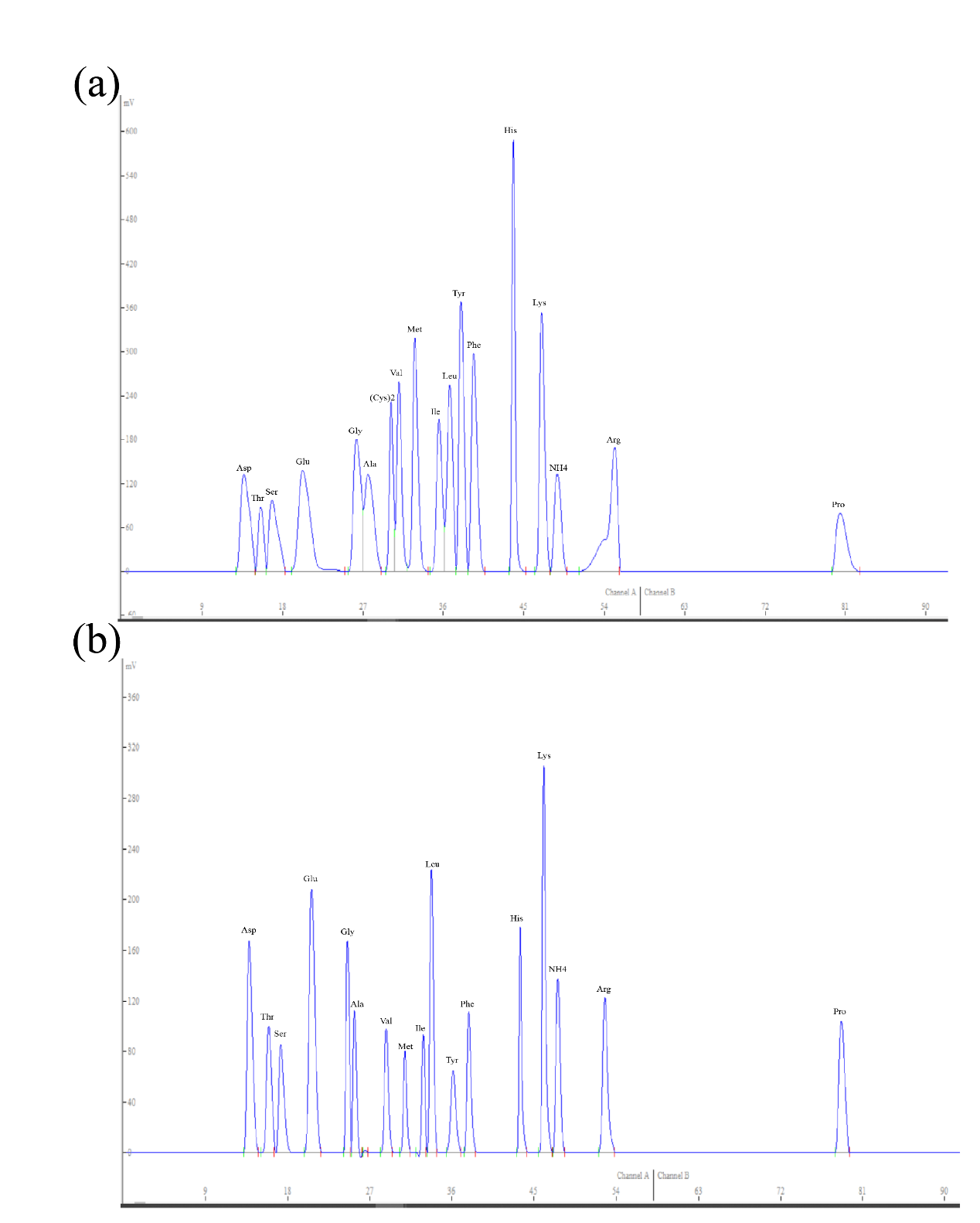
**

**Figure S5 Amino acid peak map.** (a) represents the peak diagram of amino acid standards. (b) represents the amino acid peak diagram of the sample. Asp, Asparagine; Thr, Threonine; Ser, Serine; Glu, Glutamate; Gly, Glycine; Ala, Alanine; Val, Valine; Met, Methionine; Ile, Isoleucine; Leu, Leucine; Tyr, Tyrosine; Phe, Phenylalanine; Lys, Lysine; His, Histidine; Arg, Arginine; Pro, Proline.
